# WT1-mediated repression of the proapoptotic transcription factor ZNF224 is triggered by the BCR-ABL oncogene

**DOI:** 10.18632/oncotarget.4950

**Published:** 2015-07-22

**Authors:** Giorgia Montano, Karina Vidovic, Chiara Palladino, Elena Cesaro, Gaetano Sodaro, Concetta Quintarelli, Biagio De Angelis, Santa Errichiello, Fabrizio Pane, Paola Izzo, Michela Grosso, Urban Gullberg, Paola Costanzo

**Affiliations:** ^1^ Department of Molecular Medicine and Medical Biotechnology University of Naples Federico II, Naples, Italy; ^2^ Department of Haematology and Transfusion Medicine, BioMedical Center, Lund University, Lund, Sweden; ^3^ Department of Clinical Medicine and Surgery, University of Naples Federico II, Naples, Italy

**Keywords:** ZNF224, chronic myeloid leukemia, BCR-ABL, WT1, tyrosine kinase inhibitors

## Abstract

The Kruppel-like protein ZNF224 is a co-factor of the Wilms’ tumor 1 protein, WT1. We have previously shown that ZNF224 exerts a specific proapoptotic role in chronic myelogenous leukemia (CML) K562 cells and contributes to cytosine arabinoside-induced apoptosis, by modulating WT1-dependent transcription of apoptotic genes. Here we demonstrate that ZNF224 gene expression is down-regulated both in BCR-ABL positive cell lines and in primary CML samples and is restored after imatinib and second generation tyrosine kinase inhibitors treatment. We also show that WT1, whose expression is positively regulated by BCR-ABL, represses transcription of the ZNF224 gene. Finally, we report that ZNF224 is significantly down-regulated in patients with BCR-ABL positive chronic phase-CML showing poor response or resistance to imatinib treatment as compared to high-responder patients. Taken as a whole, our data disclose a novel pathway activated by BCR-ABL that leads to inhibition of apoptosis through the ZNF224 repression. ZNF224 could thus represent a novel promising therapeutic target in CML.

## INTRODUCTION

The Kruppel-like zinc-finger protein ZNF224 was initially identified as the transcriptional repressor of the human aldolase gene [1]. Similarly to other zinc finger proteins containing the KRAB repressor domain, ZNF224 recruits, through its KRAB domain, the KAP1 co-repressor complex containing histone deacetylases and enzyme activities modifying chromatin to repress gene transcription [2, 3]. The arginine methyltransferase type II, PRMT5, was identified as an additional component of the ZNF224 transcriptional repression complex, which mediates methylation of arginine 3 of histone H4 in the nucleosomes surrounding the promoter region, thus eliciting the repression of gene expression [4].

More recently, our findings have highlighted the role of ZNF224 as a transcriptional co-factor of the Wilms’ tumour protein 1, WT1, that is achieved through the interaction of ZNF224 with the WT1(−KTS) isoform [5]. WT1 plays an oncogenic role in a wide range of solid tumors and hematopoietic malignancies, including chronic myelogenous leukemia (CML) [6-8]. More in detail, WT1 exerts anti-apoptotic functions in leukemic cells by controlling the expression of several apoptotic genes, including proapoptotic bcl-2 family members [9, 10] and the anti-apoptotic genes A1/Bfl-1 [11] and bag3 [12]. In a previous study we were able to demonstrate that ZNF224 acts as a co-activator of WT1(−KTS) in the regulation of proapoptotic genes and suppresses WT1-mediated transactivation of antiapoptotic genes in the CML-derived cell line K562, thus pointing to a role for ZNF224/WT1(−KTS) interaction in leukemia. We also demonstrated that ZNF224 plays a relevant role in ara-C-induced apoptosis of leukemia cells [13].

CML is a myeloproliferative disorder characterized by the *BCR-ABL* gene rearrangement. The BCR-ABL oncoprotein possesses an ABL tyrosine kinase domain that is constitutively activated [14] and supports malignant trasformation by activating multiple signal transduction pathways that promote uncontrolled cell proliferation [15], abnormal cell adhesion [16] and resistance to many apoptotic stimuli induced by antileukemic drugs [17, 18]. Nevertheless, the antiapoptotic pathways triggered by BCR-ABL are still poorly understood.

Our previous findings prompted us to investigate the effects of imatinib and second generation tyrosine kinase inhibitors (TKIs) dasatinib and nilotinib on ZNF224 expression levels and to identify the molecular mechanisms of ZNF224 down-regulation in CML cells. In this study we demonstrate that inhibition of BCR-ABL tyrosine kinase activity, induced by imatinib, triggers the up-regulation of ZNF224 expression at the transcriptional level. Moreover, we show that WT1 is involved in the transcriptional repression of ZNF224 in BCR-ABL expressing cells, in accordance with a recent finding indicating that WT1 is a BCR-ABL survival factor and its expression is induced via the phosphatidylinositol-3 kinase (PI3K)-Akt pathway [19]. Finally, we found a correlation between ZNF224 mRNA expression levels and responsiveness to imatinib therapy in patients with BCR-ABL positive chronic phase CML (CP-CML). This suggests that ZNF224 could be exploited as a novel predictive factor for imatinib response in CML patients.

## RESULTS

### ZNF224 expression is down-regulated in BCR-ABL positive cell lines and CD34+ primary cells derived from CML patients

**Figure 1:**
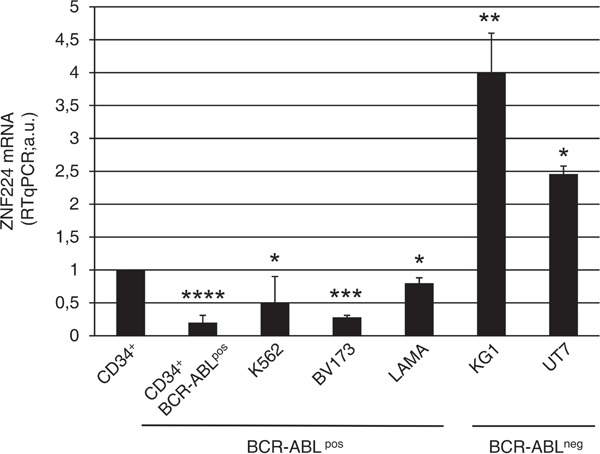
ZNF224 expression in CD34+ primary bone marrow cells from CML patients and in human myeloid leukemia cell lines Quantitative evaluation by RT-qPCR of ZNF224 mRNA expression in CD34+ primary cells collected from 10 CML patients, in BCR-ABL^pos^ cell lines (K562, BV173, LAMA84), or in BCR-ABL^neg^ cell lines (KG1, UT7). mRNA levels of normal human CD34^+^ cord blood cells were referred to as 1. Error bars represent standard deviations of two independent experiments. ****P = 0.0000017, ***p = 0.00006, **p = 0.0097, *p < 0.02.

To address whether BCR-ABL expression is associated with down-regulation of ZNF224, we initially measured ZNF224 mRNA levels in leukemia cell lines (K562, BV173, LAMA84) derived from CML patients, in CD34+ primary bone marrow cells derived from 10 CML patients at diagnosis, all characterized by the presence of BCR-ABL fusion gene, or in BCR-ABL negative cell lines (KG1, UT7) derived from patients with acute myeloid leukemia (AML). As shown in Figure 1, the expression levels of ZNF224 were significantly lower in BCR-ABL positive cell lines as well as in CD34+ primary cells from CML patients with respect to BCR-ABL negative cell lines.

### TKIs induce expression of ZNF224 in BCR/ABL positive cell lines

**Figure 2:**
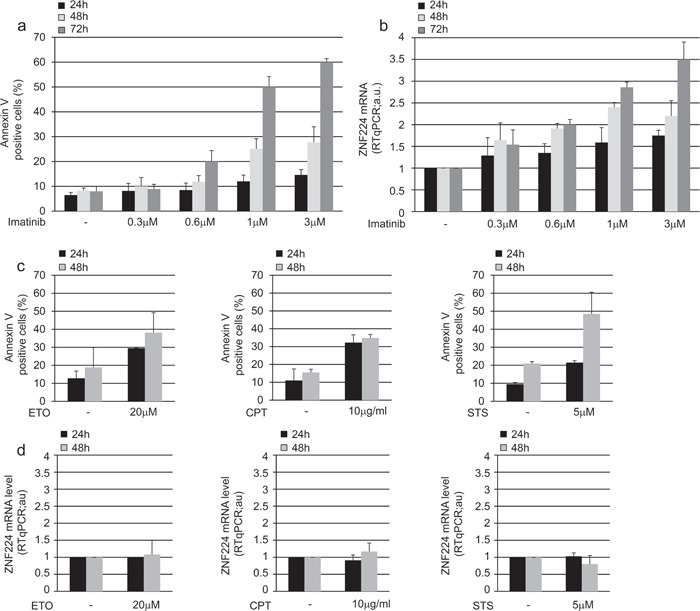
ZNF224 expression in drug-treated K562 cells K562 cells were exposed to increasing concentration of imatinib (0.3 μM, 0.6 μM, 1 μM, 3 μM) or vehicle only (DMSO) as control and analyzed after 24, 48 and 72h. **a.** Apoptosis was determined by annexin V-APC staining followed by flow cytometry. Results represent the mean +/− SD of three independent experiments. **b.** ZNF224 mRNA levels were measured by RT-qPCR. Relative amounts as compared to control are shown. Error bars represent standard deviations of three independent experiments. **c. d.** K562 cells were treated with 20 μM Etoposide (ETO), 10 μg/ml Camptothecin (CPT), 5 μM Staurosporine (STS) or vehicle only (DMSO) as control and analyzed for apoptosis as determined by Annexin V-APC staining followed by flow cytometry (c) and ZNF224 mRNA levels as determined by RT-qPCR analysis (d) after 24 and 48h. Results represent the mean +/− SD of two independent experiments.

To investigate the functional activity of BCR-ABL on ZNF224 expression, we treated K562 cells with increasing concentrations of the tyrosine kinase inhibitor imatinib for 24, 48 and 72 h, after which annexin assay was performed to evaluate apoptosis, and ZNF224 mRNA levels were measured (Figure 2). As expected, annexin positivity was induced by imatinib in a dose and time-dependent manner (Figure 2a); interestingly, we observed that exposure of K562 cells to imatinib also resulted in a time and dose-dependent up-regulation of ZNF224 mRNA expression (Figure 2b). To evaluate whether ZNF224 expression was selectively induced by BCR-ABL inhibition, thus excluding that it occurred as consequence of apoptotic machinery activation, we treated K562 cells with topoisomerase inhibitors etoposide and camptothecin and with a PKC inhibitor, staurosporine. As expected, treatment with each of these three drugs induced apoptosis, as revealed by the increased annexin-V binding (Figure 2c), whereas no upregulation of ZNF224 expression was observed (Figure 2d), thus indicating that ZNF224 expression is specifically related to BCR-ABL-inhibition.

**Figure 3:**
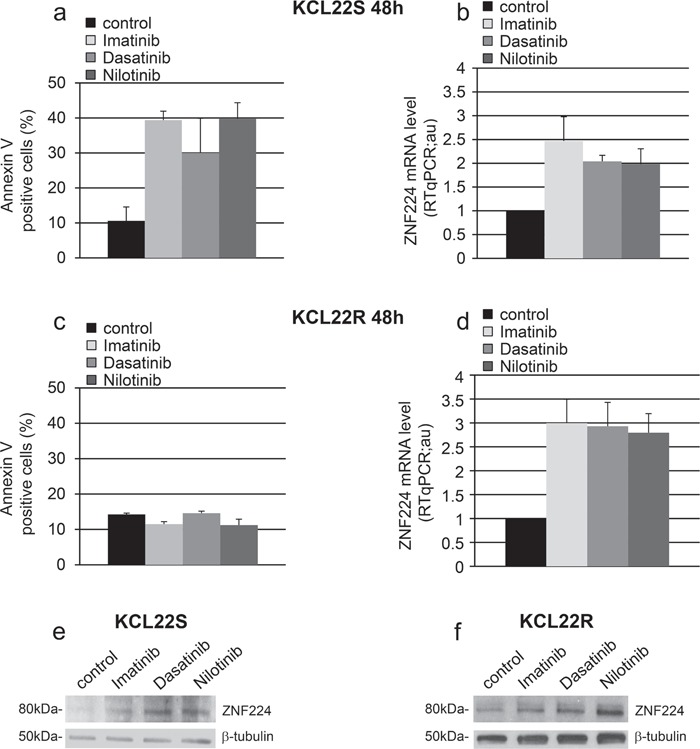
Effects of imatinib and second-generation TKIs on ZNF224 expression in KCL22 CML cell lines KCL22S (a, b, e) and KCL22R (c, d, f) cells were treated with 5 μM imatinib, 0.03 μM dasatinib or 0.4 μM nilotinib or vehicle only (DMSO) as control for 48h. **a.**, **c.** Apoptosis was determined by annexin V-APC staining followed by flow cytometry. Results represent the means +/− SD of two independent experiments. **b.**, **d.** ZNF224 mRNA levels were measured by RT-qPCR. Error bars represent standard deviations of two independent experiments. **e.**, **f.** Western blot analysis of ZNF224 in KCL22S (e) and KCL22R (f). β-tubulin was used as loading control. Molecular weight to the left. One representative blot out of two performed is shown.

To provide additional evidence that BCR-ABL signaling represses ZNF224 expression we used the BCR-ABL^pos^ cell line KCL22-S and its imatinib-resistant counterpart KCL22-R. These resistant cells are no longer dependent on oncogenic BCR-ABL kinase activity for survival, and thus imatinib at high concentration (5 μM) suppresses BCR-ABL activity, without affecting their viability [20]. KCL22-S and KCL22-R cells were treated with 5 μM imatinib for 48h after which apoptosis and ZNF224 expression were analyzed. As expected, imatinib was able to induce annexin positivity only in the sensitive (Figure 3a), and not in the resistant KCL22 cell line (Figure 3c). On the contrary, imatinib was able to induce ZNF224 mRNA expression (Figure 3b and 3d) and ZNF224 protein (Figure 3e and 3f) in both cell lines, correlating to suppression of BCR-ABL activity in both sensitive and resistant cells [20]. We then investigated whether ZNF224 mRNA expression is also modulated by the second-generation TKIs, dasatinib and nilotinib. To this aim, we exposed KCL22-S and KCL22-R cell lines to either dasatinib or nilotinib (0.03 μM and 0.4 μM, respectively) for 48 h. We found that both compounds were able to induce apoptosis only in the sensitive but not in the resistant cell line (Figure 3a and 3c), whereas, similarly to the imatinib treatment, ZNF224 mRNA expression was induced in both KCL22-S and KCL22-R cell lines (Figure 3b and 3d). Western blot analysis confirmed the induction of ZNF224 protein by dasatinib and nilotinib in both KCL22-S and KCL22-R cells (Figure 3e and 3f). These findings further strengthen the conclusion that increased ZNF224 expression follows inhibition of BCR-ABL signaling, rather than being a non-specific consequence of apoptosis induction. It is to be noted that inhibition of the BCR-ABL kinase activity and the resulting increase of ZNF224 in the resistant KCL22-R cell line are not sufficient to induce apoptosis, thus indicating that concomitant mechanisms, hampering the expected cell death increase, are responsible for the resistance to apoptosis in these cells.

### Expression of BCR-ABL in KG1 cell line down-regulates ZNF224 expression

**Figure 4:**
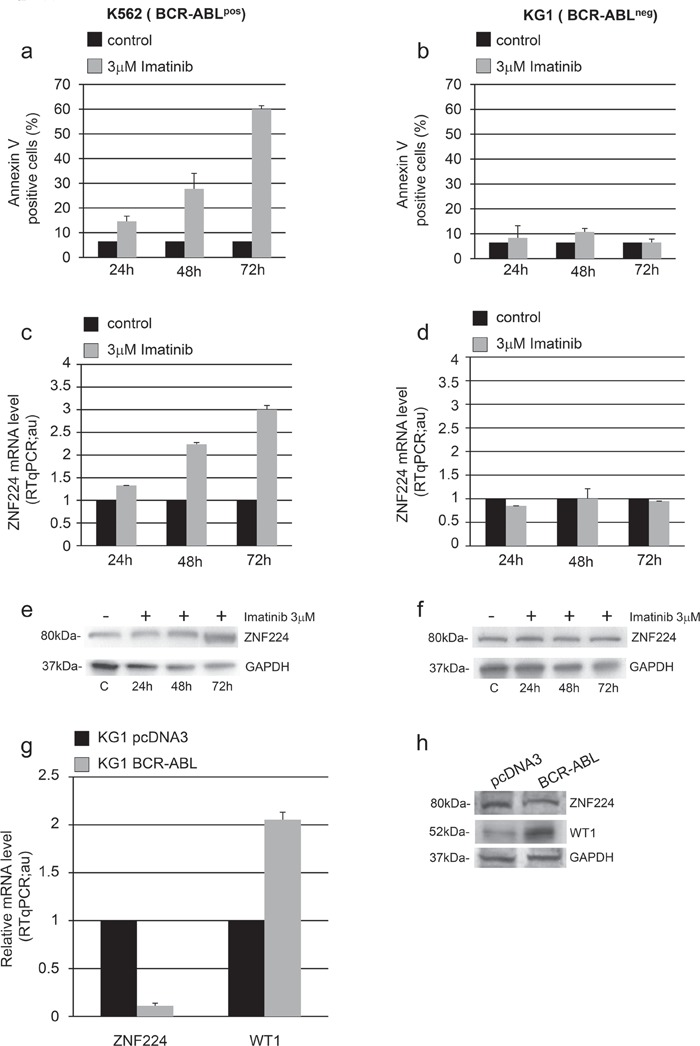
Role of BCR-ABL in ZNF224 down-modulation BCR-ABL^pos^ K562 cells (a, c, e) and BCR-ABL^neg^ KG1 cells (b, d, f) were exposed to 3 μM imatinib or vehicle only (DMSO) as control for 24, 48 and 72h. **a.**, **b.** Apoptosis was determined by annexin V-APC staining followed by flow cytometry. Results represent the mean +/− SD of two independent experiments. **c.**, **d.** Quantitative evaluation by RT-qPCR of ZNF224 mRNA expression in K562 and KG1 cells. Error bars represent standard deviations of two independent experiments. **e.**, **f.** Western blot analysis of ZNF224 in K562 and KG1 cells; C: cells treated with DMSO for 72h used as control. GAPDH was used as loading control. One representative blot out of two performed is presented. **g.** Quantitative evaluation by RT-qPCR of ZNF224 and WT1 mRNA expression levels in KG1 cells transfected for 48h with BCR-ABL expression vector or pcDNA3 empty vector as control. Error bars represent standard deviations of two independent experiments. **h.** Western blot analysis of ZNF224 and WT1 in KG1 cells transfected for 48h with BCR-ABL expression vector or pcDNA3 empty vector as control. GAPDH was used as loading control. Molecular weight to the left. One representative blot out of two performed is presented.

To confirm that ZNF224 increase is specifically related to BCR-ABL inhibition, we exposed K562 (BCR-ABL^pos^) and KG1 (BCR-ABL^neg^) cells to 3 μM imatinib for 24, 48 and 72h. As expected, imatinib-treatment induced apoptosis in BCR-ABL positive K562 cells (Figure 4a), but not in the BCR-ABL negative KG1 cells (Figure 4b). Similarly, ZNF224 induction both at mRNA and protein level was observed only in imatinib-treated K562 cells (Figure 4c and 4e) and not in imatinib-treated KG1 cells (Figure 4d and 4f).

To definitively prove the role of BCR-ABL in down-regulation of ZNF224 expression, we transfected KG1 cells with an expression vector encoding human BCR-ABL and with empty vector as control. Ectopic expression of BCR-ABL in KG1 strongly reduced ZNF224 both at mRNA (Figure 4g) and protein levels (Figure 4h). As shown, a considerable increase in WT1 expression was observed in response to BCR-ABL (Figure 4g and 4h), consistent with a previous study showing that the tyrosine kinase activity of BCR-ABL signals increases expression of WT1 (19).

All together, these data demonstrate that imatinib could hamper a BCR-ABL signaling-dependent mechanism of ZNF224 repression.

### Imatinib enhances ZNF224 mRNA expression via transcriptional activation

**Figure 5:**
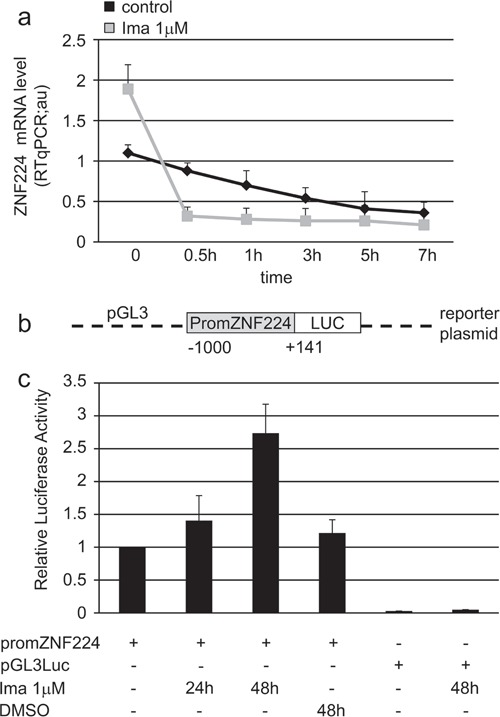
Imatinib enhances ZNF224 mRNA expression via transcriptional mechanism **a.** K562 cells were treated with 1 μM imatinib or vehicle only (DMSO) as control for 48h after which 4 μg/ml actinomycin D was added to block *de novo* mRNA synthesis. ZNF224 mRNA levels were determined by RT-qPCR 0.5, 1, 3, 5 and 7h after addition of actinomycin D. Error bars represent standard deviations of three independent experiments. **b.** Schematic representation of the reporter plasmid containing the ZNF224 promoter region (−1000 to +141) used in transient transfection experiments (promZNF224). **c.** K562 cells were transfected with promZNF224 for 24h, after which cells were incubated with 1 μM imatinib for 24 and 48h or vehicle only (DMSO) as control, for 48h. The promoter activity was measured by normalizing firefly to renilla luciferase activity. pGL3Luc null empty vector activity indicates the background. Data shown are the mean values +/−S.D. of three independent experiments, each of which included triplicate determinations.

We next determined whether the induction of ZNF224 mRNA expression by imatinib is a result of altered ZNF224 mRNA stability. K562 cells were incubated for 48 h with or without 1 μM imatinib after which actinomycin D was added to block *de novo* mRNA synthesis. After addition of actinomycin D, the decay of ZNF224 mRNA was monitored by repeated real time RT-PCR analyses. Pretreatment with imatinib did not increase the stability of ZNF224 transcript rather it was slightly decreased, as indicated by a somewhat more rapid decline following actinomycin D addition (Figure 5a). The same analysis was performed in K562 cells incubated with 1μM imatinib for 24h (data not shown). These results indicate that imatinib modulates ZNF224 mRNA expression by transcriptional activation of the ZNF224 gene. To demonstrate transcriptional effects, K562 cells were transiently transfected with the ZNF224 promoter cloned in a luciferase reporter plasmid (promZNF224) (Figure 5b), and then treated with 1 μM imatinib for 24 and 48h. As shown in Figure 5c, the basal ZNF224 promoter activity was increased upon exposure to imatinib. In contrast, imatinib showed no effect on pGL3-control promoter (Figure 5c). On the basis of these experiments, we conclude that imatinib, via inhibition of BCR-ABL tyrosine kinase activity, increases ZNF224 expression through a transcriptional mechanism.

### WT1 represses ZNF224 gene expression

**Figure 6:**
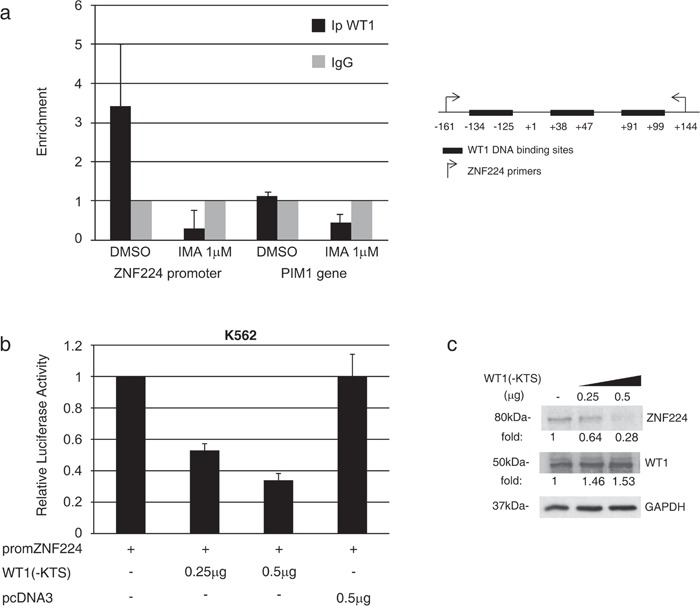

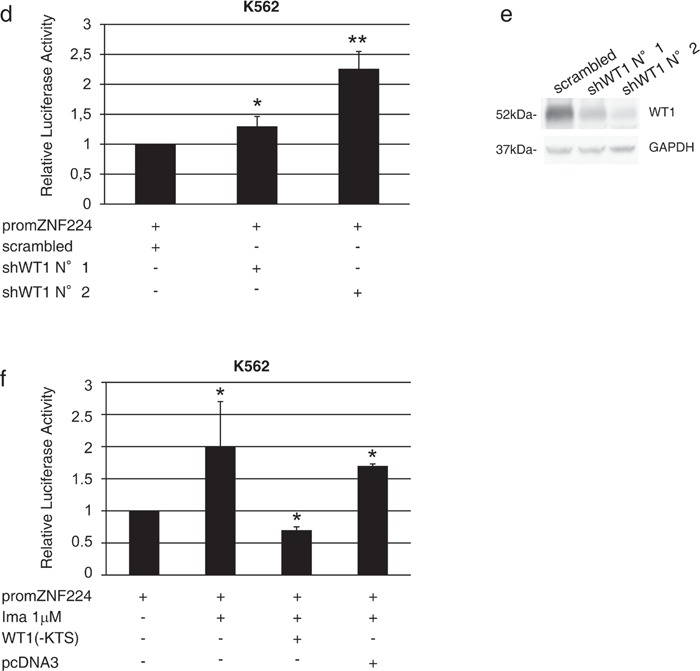
WT1(−KTS) binds to the ZNF224 promoter and represses ZNF224 expression **a.** K562 cells were treated with 1 μM imatinib or vehicle only (DMSO) as control for 24h after which chromatin immunoprecipitation assay was performed with an anti-WT1 antibody (C19). Immunoprecipitation with non-specific IgG was used as negative control. Quantitative real time PCR analysis was performed using specific ZNF224 primers, covering the 3 putative WT1 binding sites on ZNF224 promoter region (−161 to +144). PIM1 was used as negative control. Error bars indicate the mean value +/− SD of two independent experiments. **b.** K562 cells were transfected with promZNF224 together with 0.25 μg and 0.5 μg of WT1(−KTS) expression plasmid or with 0.5 μg of empty pcDNA3 as control. After 48h, promoter activity was determined by normalizing firefly to renilla luciferase activity. Shown is promoter activity relative to that of promZNF224 in control cells (mean values +/−S.D., three independent experiments). **c.** Western blot analysis of ZNF224 and WT1 proteins in cell lysates of K562 cells transfected with 0.25 μg or 0.5 μg of WT1(−KTS) expression plasmid for 48h. GAPDH was used as loading control. Molecular weights to the left. One representative result out of two performed is presented. **d.** K562 cells transduced with two different shRNAs targeting WT1 (shWT1 N°1 and shWT1 N°2) or a scrambled control were transfected with promZNF224 for 48h. Promoter activity was determined by normalizing firefly to renilla luciferase activity. Shown is promoter activity relative to that of promZNF224 in cells transduced with a scrambled control (mean values +/−S.D., 3 independent experiments). *P = 0.03; **P = 0.0006. **e.** Western blot analysis of WT1 protein levels in K562 cells transduced with shWT1 N°1, shWT1 N°2 or a scrambled control. GAPDH was used as loading control. **f.** K562 cells were transfected with promZNF224 with or without 0.5 μg of WT1(−KTS) expression plasmid or 0.5 μg of pcDNA3 as control. 24h after transfection, the cells were incubated for 48 h in the presence of 1 μM imatinib, after which promoter activity was measured by normalizing firefly to renilla luciferase activity. Shown is promoter activity relative to that of promZNF224 in control cells (mean values +/−S.D., three independent experiments) *p* < 0.05.

Subsequently, to search for transcription factors potentially involved in ZNF224 regulation downstream of BCR-ABL signaling, we conducted an *in silico* analysis of putative transcription factor binding sites located in the ZNF224 promoter. Interestingly, three WT1 binding sites surrounding the ZNF224 transcription start site were found. According to the fact that BCR-ABL induces WT1 expression [19] whereas ZNF224 is down-modulated by BCR-ABL (see Figure 3), we evaluated whether WT1 could be involved in the transcriptional suppression of ZNF224 mediated by BCR-ABL. To verify WT1 occupancy on the ZNF224 promoter and the effects of imatinib on this binding, we conducted Chromatin immunoprecipitation assays (ChIP) in K562 cells incubated for 24h in the presence or absence of imatinib. Chromatin was immunoprecipitated with a WT1 antibody and real time PCR analysis was performed using oligonucleotides flanking the WT1-binding sites on the ZNF224 promoter. Results confirmed *in vivo* WT1 occupancy on the ZNF224 promoter that is abolished after imatinib treatment (Figure 6a).

To investigate the role of WT1 binding on the ZNF224 promoter, we introduced the promZNF224 reporter plasmid into K562 cells and analyzed the luciferase activity in the presence of increasing amounts of the WT1(−KTS) expression plasmid. As shown in Figure 6b, WT1(−KTS) was able to repress ZNF224 promoter activity in a dose-dependent manner. We also observed a progressive decrease in the levels of the endogenous ZNF224 protein in K562 cells transfected with increasing amounts of WT1(−KTS) (Figure 6c). The transcriptional repression exerted by WT1 on promZNF224 was confirmed by RNAi-mediated silencing of WT1. K562 cells were transduced with two different lentiviral shRNAs targeting WT1 or a scrambled control. Following transfection with promZNF224 we found that WT1 knockdown leads to a considerable increase in promZNF224 luciferase activity (Figure 6d).

Finally, to evaluate the role of WT1 on the imatinib-mediated induction of ZNF224 promoter activity, K562 cells were transfected with promZNF224 alone or co-transfected with the expression vector for WT1(−KTS) and treated with imatinib for 48h. As shown in Figure 6f, WT1 over-expression effectively counteracts the induction of ZNF224 promoter activity elicited by imatinib. These results, in agreement with ChIP data, provide further evidence that WT1 is an effector of the ZNF224 transcriptional repression induced by BCR-ABL.

### BCR-ABL-mediated suppression of ZNF224 occurs via the PI3K-Akt signaling pathway

**Figure 7:**
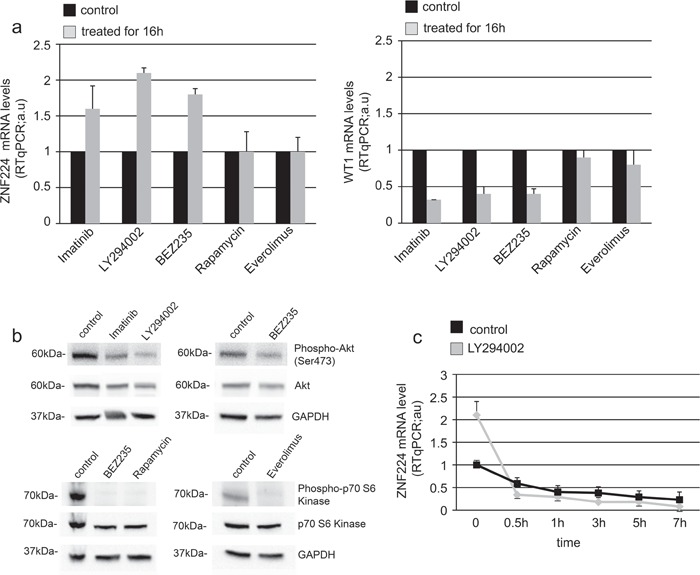
BCR-ABL decreases ZNF224 expression via PI3K-Akt signaling pathway **a.** K562 cells were treated for 16h with 1 μM Imatinib, or 30 μM PI3K inhibitor (LY294002), or 20 μM dual PI3K/AKT/mTOR inhibitor (BEZ235), or 50 ng/ml mTOR inhibitor (Rapamycin) or 1 μM mTOR inhibitor (Everolimus) or vehicle only (DMSO) as control. ZNF224 and WT1 mRNA levels were measured by RT-qPCR. Error bars represent standard deviations of three independent experiments. **b.** Protein extracts were subjected to Western blotting with antibodies against phospho-Akt (Ser473), total Akt, phospho-p70 S6-kinase and total p70 S6-kinase. GAPDH was used as loading control. One representative blot out of two performed is presented. Molecular weights to the left. **c.** K562 cells were incubated for 16h with 30 μM LY294002 or vehicle only (DMSO) as control, after which 4 μg/ml actinomycin D was added to block *de novo* mRNA synthesis. Cells were collected at 0, 0.5, 1, 3, 5 and 7h after addition of actinomycin D and RT-qPCR analysis of ZNF224 mRNA levels was performed. Error bars represent standard deviations of three independent experiments.

The phosphatidylinositol-3 kinase (PI3K)-Akt pathway is one of the three major signaling pathways constitutively activated by the BCR-ABL tyrosine kinase activity [21, 22] and it has been shown to mediate the BCR-ABL-induced upregulation of WT1 expression [19]. Therefore, we expected that also suppression of ZNF224 by BCR-ABL should be dependent on the PI3K-Akt pathway. To investigate this issue, K562 cells were treated for 16 h either with imatinib, PI3K inhibitor (LY294002), dual PI3K/AKT/mTOR inhibitor (BEZ235) or with the mTOR inhibitors Rapamycin and Everolimus, after which ZNF224 expression was evaluated (Figure 7a, left panel). In agreement with data shown in Figure 2b, imatinib increased the levels of ZNF224. Interestingly, ZNF224 mRNA increased following inhibition of PI3K or Akt, while mTOR inhibition did not affect ZNF224 mRNA expression, thus indicating the involvement of PI3K-Akt signaling pathway in ZNF224 suppression. According to previous results [19], we observed that inhibition of PI3K or Akt, but not mTOR, resulted in a decrease of WT1 mRNA (Figure 7a, right panel). To confirm the inhibition of the PI3K pathway by LY294002 and BEZ235, phosphorylation of Akt was analyzed by Western blot; phosphorylation of p70S6-kinase (a substrate phosphorylated by mTOR) was analyzed to confirm the efficacy of BEZ235, Rapamycin and Everolimus (Figure 7b). Furthermore, by inhibiting mRNA synthesis with actinomycin D we showed that PI3K signaling did not significantly affect the ZNF224 mRNA stability (Figure 7c), as already observed after treatment with imatinib (Figure 5a).

These data provide evidence that BCR-ABL-mediated suppression of ZNF224 expression is dependent on the PI3K pathway, via the transcriptional repression exerted by WT1 on ZNF224 gene expression.

### ZNF224 expression in patients with chronic myeloid leukemia

**Figure 8:**
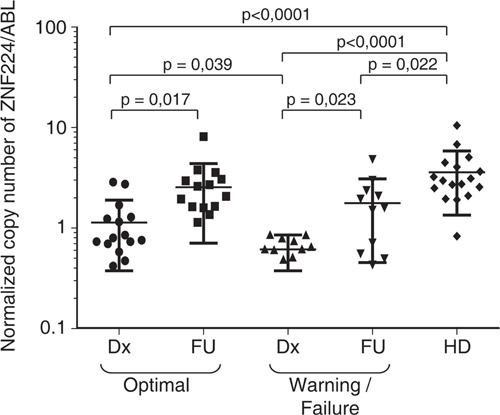
Evaluation of ZNF224 mRNA expression level in PB samples derived from patients affected by CP-CML ZNF224 expression level assessed by RT-qPCR in PB of 30 patients with CP-CML at diagnosis (Dx) and at follow up after three months of imatinib treatment (FU). Patients were classified, according to the ENL definitions in Optimal and Warning/Failure Responder (23). Shown are levels of ZNF224 mRNA expression levels in Optimal and Warning/Failure Responder at diagnosis (Dx) and at follow up (FU), as compared to those detected in 17 healthy blood donors (HD). Strait lines indicate mean values of the five groups of subjects. Statistical analysis was performed by nonparametric Mann-Whitney test.

Finally, we analyzed ZNF224 mRNA expression in peripheral mononuclear cells from 30 adult patients with BCR-ABL positive chronic phase CML (CP-CML) at diagnosis (Dx) and after three months (FU) of treatment with imatinib standard dose as a first-line therapy. On the basis of response at 12 months of imatinib therapy the patients were clustered in optimal responders (15 patients) and warning/failure responders (15 patients), according to the criteria of Baccarani et al. [23]. Intriguingly, as shown in Figure 8, at diagnosis ZNF224 mRNA levels in both patient cohorts were significantly lower than in healthy donors (HDs) (*p* < 0.0001). Furthermore, we observed a correlation between the baseline levels of ZNF224 mRNA and imatinib responsiveness. In fact, at diagnosis optimal responders showed ZNF224 mRNA levels significantly higher than warning/failure responders (*p* = 0.039) while at follow-up ZNF224 expression was found significantly increased in all patients. These findings indicate that higher ZNF224 levels at diagnosis could correlate with better treatment responsiveness.

## DISCUSSION

The leukemogenic BCR-ABL fusion protein activates multiple signal transduction pathways which, in turn, may affect the expression of genes implicated in the pathogenesis of CML [14]. One of the key mechanisms implicated in the malignant transformation by BCR-ABL is represented by inhibition of apoptosis [21], which may be triggered through the down-modulation of different proapoptotic factors [24]. Some of these have been recently shown to be indirect transcriptional targets of the BCR-ABL oncoprotein. Expression of Bim is inhibited in BCR-ABL positive cells, due to inactivation of FoxO3a, a trans-activator of Bim. Imatinib treatment results in FoxO3a activation, induction of Bim expression and apoptosis [25]. Likewise, it has been recently demonstrated that BCR-ABL represses at transcriptional level the lymphoid-specific transcription factor BACH2 through the suppression of Pax5 expression, a potent trans-activator of BACH2 [26].

We previously showed that the zinc finger protein ZNF224 is crucial for ara-C induced apoptosis of CML cells. The pro-apoptotic effect of ZNF224 results mainly from its role as a cofactor for the transcription factor WT1; more in detail, we found that ZNF224 induction by ara-C enhances apoptosis in K562 cells through the down-regulation of antiapoptotic WT1 target genes such as bag3 and A1/Bfl1 and the upregulation of proapoptotic genes such as VDR, Bax and Bak [13]. Here we show that ZNF224 expression is down-regulated in BCR-ABL positive CML cell lines and primary CD34-enriched cells from CML patients. More interestingly, imatinib and second-generation TKIs nilotinib and dasatinib are able to specifically restore ZNF224 expression. By investigating the molecular mechanisms involved in the imatinib-dependent ZNF224 induction, we demonstrate that ZNF224 is an indirect transcriptional target of BCR-ABL. Furthermore, we have identified WT1, whose expression is induced by the oncogenic signaling triggered by BCR-ABL via the PI3K/AKT pathway [19], as a transcriptional repressor of ZNF224 in CML. Similarly, WT1 mediates BCR-ABL induced repression of the myeloid tumor suppressor IRF8 [27].

Data herein presented regarding the role of WT1 as a repressor of ZNF224 gene in CML could also contribute to explain the mechanism leading to ZNF224 induction by ara-C and the consequent apoptosis; indeed, our previous observation that ara-C induces a down-modulation of WT1 in K562 cells [13] correlates well with the ZNF224 up-regulation that we found following treatment with either ara-c or imatinib. Our data, thus, lead us to hypothesize that the distinct signaling cascades induced by ara-C and imatinib could converge on ZNF224 induction through the down-modulation of WT1. Therefore, the current study represents a further advancement with respect to our previous findings [13] and could contribute to shed light on the mechanisms of ara-C induced apoptosis, that are still largely undetermined.

In addition, our molecular data are suggestive of a regulatory loop in which WT1, by repressing the expression of ZNF224 gene, could prevent the proapoptotic effect of the ZNF224-WT1 complex itself, thus contributing to the WT1 pro-survival role in leukemia.

Similarly to our results on WT1 and ZNF224, a regulatory circuit was proposed between WT1 and Pax-2 in kidney development in which WT1 protein has been shown to inhibit the expression of Pax-2 gene [28] that, in turn, acts as a transcriptional activator of the WT1 gene [29]. Furthermore, WT1 and Pax-2 proteins are able to form a molecular complex, that could affect the transcriptional regulatory properties of the two proteins [30]. The identification of ZNF224 as a downstream target of BCR-ABL kinase activity may lead to important new insights in the molecular mechanisms underlying CML. Particularly, the development of imatinib resistance in CML patients is a dramatic issue in clinical practice, and a key topic currently addressed in CML research. Amplification of the BCR-ABL gene or point mutations within the BCR-ABL kinase domain are the most commonly identified mechanisms of resistance to imatinib [31, 32]. Our data strongly suggest that the induction of ZNF224 expression could provide promising means to circumvent imatinib resistance and to develop new therapeutic approaches in CML.

Finally, preliminary data from CML patients suggest that ZNF224 could be a novel biomarker at diagnosis to predict the imatinib sensitivity in patients with chronic myelogenous leukemia, although analysis of ZNF224 expression levels in a larger cohort of patients is required in order to assess its potential use in clinical practice.

## MATERIALS AND METHODS

### Cell lines

HEK293T/17 human cell lines were cultured in Dulbecco’s modified Eagle’s medium (Bio-Whittaker, Verviers, Belgium) supplemented with 10% fetal calf serum, 100 μg/ml streptomycin-penicillin mix (Bio-Whittaker) at 37°C in 5% CO2.

Acute and chronic myeloid leukemia cell lines were cultured in RPMI 1640 supplemented with 10% fetal calf serum and 100 μg/ml penicillin-streptomycin mix (Bio-Whittaker) at 37°C in 5% CO2. The KCL22-R cell line was further supplemented with 1μM imatinib (kindly provided by Novartis Pharma, Basel, Switzerland) every 48 h [20].

### Reagents

K562 cells were treated with 20 μM etoposide (Sigma-Aldrich, St Louis, MO, USA), 10 μg/ml camptothecin (Sigma-Aldrich), 5 μM staurosporine (Sigma-Aldrich).

The KCL22-S and KCL22-R cell lines were incubated in the presence of either 5 μM imatinib, 400 nM nilotinib (Novartis Pharma), or 3 nM dasatinib (Bristol-Myers Squibb Company Princeton, New Jersey, USA).

To inhibit different signaling pathways, K562 cells were treated with either 1 μM imatinib (Novartis Pharma), 30 μM LY294002 (Calbiochem, Merck KgaA, Darmstadt, Germany), 10 μM NVP-BEZ235 (Novartis Pharma), 50 ng/ml rapamycin (Cell Signalling Technology, Beverly, MA, USA) or 1 μM everolimus (Sigma-Aldrich). To investigate ZNF224 mRNA stability, cells were treated with 4 μg/ml actinomycin D (Sigma-Aldrich).

### Samples from CML patients and healthy donors

After ethical approval and informed consent, umbilical cord blood samples from mothers giving birth to normal, full term infants, and bone marrow (BM) aspirates from 10 adult patients with BCR-ABL positive CP-CML at diagnosis, were collected after signed informed consent. CD34+ cells were enriched as described [33, 34]. CD34+ cell purity was always more than 90% as determined by flow cytometric analysis (data not shown). Peripheral blood (PB) mononuclear cells from 30 adult patients with BCR-ABL positive chronic phase chronic myeloid leukaemia (CP-CML) and 17 healthy donors (HDs) were collected after approval by the Institutional Review Board (IRB) of the University of Naples Federico II. Patients were enrolled from July 2012 in the Division of Hematology at University of Naples Federico II, and treated with the standard dose of imatinib (400 mg/d) as a first-line therapy. In particular, for retrospective analysis, we selected 15 consecutive optimal responding patients and 15 warning/failure patients [23]. PB samples were available from the patients at diagnosis and at the third month of therapy.

### RNA isolation, reverse transcription and real-time PCR

Total RNA was isolated using the RNeasy Mini Kit (Qiagen, Hilden, Germany) according to the manufacturer’s protocol. 1 μg of RNA was reverse transcribed using the QuantiTect Reverse Transcription Kit (Qiagen), according to the manufacturer’s protocol. To analyze the expression levels of ZNF224 mRNA in K562 cells treated with imatinib, etoposide, camptothecin, staurosporine, and actinomycin D and in KCL22-S and KCL22-R cells treated with imatinib, dasatinib and nilotinib, real-time PCR was carried out in a Real-Time CFX 69 System (Bio-Rad, Berkeley, CA, USA) using the SYBR Green I Master Mix (Bio-Rad) and specific primers for ZNF224 [13]; β2-microglobulin (primer sequences: Fw 5′-ccgtggccttagctgtgct-3′, Rev 5′-tcggatggatgaaacccaga-3′) was used as reference gene for relative quantification.

TaqMan probe-based chemistry (Applied Biosystems) was used in other RT-qPCR experiments to evaluate the expression levels of ZNF224 and WT1. Probes for ZNF224 (Hs00273760) and for WT1 (Hs00240913) were purchased as Assay-on-Demand (Applied Biosystems). The amplification reactions were all performed in triplicates on an ABI Prism 7000 Sequence Detection System (Applied Biosystems). Data were collected and analyzed using the Sequence Detector v.1.1 software (Applied Biosystems). β2-microglobulin (Hs99999903) was used as reference gene. The relative quantification of gene expression was determined using the ΔΔCT method [35].

For PB samples from CP-CML patients and healthy blood donors, BCR-ABL mRNA was evaluated as standardized within the framework of the Europe Against Cancer Program [36]. ABL (NM005157.3) was used as housekeeping control gene. Results were expressed as percent ratio of BCR-ABL/ABL on the International Scale (IS) using a laboratory-specific conversion factor (CF) [37-39]. All reactions were amplified in triplicate on a 7900HT Fast Real-Time PCR System (Applied Biosystems).

### Statistical analysis

All data are presented as mean±SD. The Student’s t test was used to evaluate the statistical significance of differences using the non parametric Mann-Whitney test, with a *p* value < 0.05 indicating a significant difference.

### Cloning of the ZNF224 promoter

A 1000 bp fragment of the human ZNF224 proximal promoter was amplified from human genomic DNA using the KpnI Fw primer: 5′-ggggtaccccgttgcagtgagctaagatcgtgcc-3′, and the HindIII Rev primer: 5′-gacctggatgcgtaacctaggagtgggttcgaaccc-3′. The amplified fragment was cloned into KpnI/ HindIII sites of a pGL3-basic vector.

### Transient transfection and luciferase-reporter assays

KG1 cells were transiently transfected by electroporation using a Bio-Rad Gene Pulser II System (Bio-Rad). 10 μg of pcDNA3-p210BCR/ABL (kindly provided by Dr. Thoas Fioretos) or 10 μg of pcDNA3 empty vector used as control were electroporated in 9×10^6^ KG1 cells. A plasmid encoding enhanced green fluorescent protein (eGFP) was used to determine transfection efficiency. The electroporation conditions were as follows: 300 V, 960 μF, using a 0.4-cm gapped cuvette (Bio-Rad) for each condition. After 48h, cells were counted and stained for viability using trypan blue solution (Sigma-Aldrich).

K562 cells were transiently transfected using HiPerfect Reagent (Qiagen) with either 0.5 μg of a luciferase reporter plasmid containing the proximal ZNF224 promoter (PromZNF224) or with 0.5 μg of the empty pGL3-luciferase vector (pGL3-Luc), used as control. Cells were co-transfected with 0.25 or 0.5 μg of WT1(−KTS) expression plasmid, or with the pcDNA3 (0.5 μg) used as negative control. After 24h, the transfected cells were treated with either 1 μM imatinib for 24 or 48h, or with DMSO for 48h. To normalize the luciferase assays a pRL-CMV plasmid (50 ng) coding for the renilla luciferase was used. Luciferase activity was measured using the Dual-Luciferase Reporter Assay System (Promega Corporation, WI, USA) according to the manufacturer’s instructions.

### Lentiviral transduction of K562 cells

Two lentiviral shRNAs targeting WT1 pLKO.1/WT1 shRNA 1 (TRCN0000040066) (Open Biosystems, Huntsville, AL, USA) and pLKO.1/WT1 shRNA 2 (TRCN0000040067) or one GFP shRNA used as negative control (both kindly provided by A. Sweet-Cordero, Stanford University, Stanford, CA) were used for lentiviral transduction of K562 cells. Lentiviral particles were harvested after calcium phosphate transfection of HEK293T cells with the respective shRNA constructs, gag-pol and the RD114 envelope genes. For lentiviral transduction, non-tissue culture treated plates were coated with retronectin, 40 μg/ml (Takara Shuzo, Shiga, Japan) and blocked with 2% serum albumin (Sigma-Aldrich) for 30 min at room temperature. Subsequently, virus-containing medium was added and the plates were centrifuged at 1000xg for 1h at 4°C. K562 cells (400.000 cells/ml) were added to the virus coated plates and cultured at 37°C for 48h, after which 3 days of puromycin selection (1μg/ml) was performed.

### Western blot analysis

Total protein extracts were obtained as previously described [13], resolved with SDS-PAGE and transferred to Hybond membranes (Amersham Biosciences, NJ, USA). Non-specific binding sites were blocked for 2 h with 5% milk in Tris-Tween buffered saline (tTBS) (5 mM Tris pH 7.5, 15 mM NaCl, 0.1% Tween-20), washed three times with tTBS and incubated with antibodies. For the primary antibodies anti-GAPDH (7-B) (Santa Cruz Biotechnology, 1:1000), anti-β-Tubulin (Upstate, Lake Placid, NY, 1:2000), the secondary antibody goat-anti-mouse IgG (H+L)-HRP conjugated (BioRad 170-6516, 1:3000) was used. For the primary antibodies anti-WT1 (C19) (Santa Cruz Biotecnology, Santa Cruz, CA, USA, 1:500), anti-Akt (Cell Signaling Technology, Beverly, MA, USA, 1:1000), anti-Phospho-Akt Ser473 (Cell Signaling Technology, 1:1000), anti-p70 S6 Kinase (49D7) (Cell Signaling Technology, 1:1000), anti-Phospho-p70 S6 Kinase Thr389 (108D2) (Cell Signaling Technology, 1:1000), the secondary antibody goat-anti-rabbit IgG (H+L)-HRP conjugated (BioRad170-6515, 1:3000) was used. For detection, an ECL western blot detection system (Amersham Biosciences) was used. ZNF224 protein analysis was performed using the anti-ZNF224 (T3) antibody (Rabbit polyclonal antibody) (1) as previously described [13].

### Chromatin immunoprecipitation (ChIP) assay

Chromatin was immunoprecipitated with anti-WT1 (C19 Santa Cruz Biotechnology) and immunoglobulin G (IgG Sigma) as previously described [4]. Immunoprecipitated DNA was analyzed by quantitative real-time PCR using a Master Mix SYBR Green (Bio-Rad) and the following primers for ZNF224 promoter: Fw 5′-tgagtgtaatgctgcaggagtac-3′, Rev 5′-caatgcgtaggtccaggaaattc-3′; for PIM1 gene (used as negative control): Fw 5′-taaagccggggattttcagcc-3′, Rev 5′-ccgcccccatccttttact-3′. At first, we normalized the Ct value of specific antibody and control IgG with the input values (ΔCt = Ct _IpWT1 or IgG_- Ct_Input_). The fold enrichment was calculated by the ΔΔCt cycle threshold method comparing the ChIP antibody signal to a negative control IgG (Fold enrichment = ΔΔCt = 2^-(ΔCt_IpWT1_-ΔCt_IgG_). Results are representative of two independent experiments.

### Analysis of viability and apoptosis by flow cytometry

K562 cells were plated at a density of 2.5×10^5^/well in 12-well plates and were exposed to imatinib, etoposide (Sigma-Aldrich), camptothecin (Sigma-Aldrich), or staurosporine (Sigma-Aldrich). The relative amount of apoptotic cells was determined by co-staining cells with annexin V-APC (550474, BD Pharmingen, San Jose, CA, USA) and DAPI (4′,6-diamidino-2-phenylindole) (D9564, Sigma-Aldrich) as previously described (13). The percentage of cells within the population positive for DAPI and positive for annexin V was determined by analysis on a FACS Aria flow cytometer (BD Biosciences Immunocytometry System). Apoptosis of KCL22-S and KCL22-R cells was induced by imatinib, nilotinib, or dasatinib. Apoptotis rates were evaluated by Annexin-V Kit (BD Biosciences), following the manufacturer’s instructions. Cells were analyzed with a FACS Calibur (BD Biosciences). Cell number was counted in a Bürker chamber and viability was determined by trypan blue exclusion.
